# Construction and evaluation of hourly average indoor PM_2.5_ concentration prediction models based on multiple types of places

**DOI:** 10.3389/fpubh.2023.1213453

**Published:** 2023-08-10

**Authors:** Yewen Shi, Zhiyuan Du, Jianghua Zhang, Fengchan Han, Feier Chen, Duo Wang, Mengshuang Liu, Hao Zhang, Chunyang Dong, Shaofeng Sui

**Affiliations:** ^1^Shanghai Municipal Center for Disease Control and Prevention, Shanghai, China; ^2^Department of Environmental Health, Key Laboratory of the Public Health Safety, Ministry of Education, School of Public Health, Fudan University, Shanghai, China

**Keywords:** indoor air quality, PM_2.5_, prediction models, machine learning, random forest

## Abstract

**Background:**

People usually spend most of their time indoors, so indoor fine particulate matter (PM_2.5_) concentrations are crucial for refining individual PM_2.5_ exposure evaluation. The development of indoor PM_2.5_ concentration prediction models is essential for the health risk assessment of PM_2.5_ in epidemiological studies involving large populations.

**Methods:**

In this study, based on the monitoring data of multiple types of places, the classical multiple linear regression (MLR) method and random forest regression (RFR) algorithm of machine learning were used to develop hourly average indoor PM_2.5_ concentration prediction models. Indoor PM_2.5_ concentration data, which included 11,712 records from five types of places, were obtained by on-site monitoring. Moreover, the potential predictor variable data were derived from outdoor monitoring stations and meteorological databases. A ten-fold cross-validation was conducted to examine the performance of all proposed models.

**Results:**

The final predictor variables incorporated in the MLR model were outdoor PM_2.5_ concentration, type of place, season, wind direction, surface wind speed, hour, precipitation, air pressure, and relative humidity. The ten-fold cross-validation results indicated that both models constructed had good predictive performance, with the determination coefficients (R^2^) of RFR and MLR were 72.20 and 60.35%, respectively. Generally, the RFR model had better predictive performance than the MLR model (RFR model developed using the same predictor variables as the MLR model, R^2^ = 71.86%). In terms of predictors, the importance results of predictor variables for both types of models suggested that outdoor PM_2.5_ concentration, type of place, season, hour, wind direction, and surface wind speed were the most important predictor variables.

**Conclusion:**

In this research, hourly average indoor PM_2.5_ concentration prediction models based on multiple types of places were developed for the first time. Both the MLR and RFR models based on easily accessible indicators displayed promising predictive performance, in which the machine learning domain RFR model outperformed the classical MLR model, and this result suggests the potential application of RFR algorithms for indoor air pollutant concentration prediction.

## 1. Introduction

PM_2.5_ refers to particulate matter with an aerodynamic diameter of 2.5 μm or less, which is one of the environmental pollutants with the greatest impact on public health (1–3). Numerous epidemiological studies have shown that both long-term and short-term exposure to PM_2.5_ increases the risk of death from respiratory and cardiovascular diseases in the population (4–6). Studies have shown that for every 10 g/m^3^ increase in the average concentration of PM_2.5_ in ambient air, there is a 3.1% increase in hospital admissions and a 2.5% increase in mortality from chronic obstructive pulmonary disease (7). Furthermore, there is a 3% increase in emergency department visits for bronchial asthma (8), a 16% increase in the risk of death from ischemic heart disease, and a 14% increase in mortality from stroke (4, 9).

Currently, most relevant studies use ambient PM_2.5_ concentrations as a surrogate for human PM_2.5_ exposure without taking into account the difference between indoor and outdoor PM_2.5_ concentrations as well as the contribution of indoor PM_2.5_ exposure to actual human exposure, which limits the interpretation of their results. As most people spend at least 80% of their day indoors, and for some specific populations such as the older adults and children, this percentage is even higher (10–12). Therefore, indoor PM_2.5_ concentration is crucial for accurate PM_2.5_ exposure assessment and health risk assessment. Direct measurement of indoor PM_2.5_ concentration can provide the most accurate data; however, such practice is not easy to achieve, as it requires a lot of manpower and material resources as well as the compliance of the research participants, especially for large-scale population and/or long-term studies. When direct measurement is difficult to achieve, it is important to construct appropriate predictive models.

At present, many studies have been conducted to establish prediction models for indoor PM_2.5_ concentration (12–18), mainly involving multiple linear regression (MLR) models and random forest regression (RFR) models, which have their own advantages and disadvantages. For indoor PM_2.5_ concentration, there is still controversy about which model has a better predictive effect. In addition, the models in these studies have mostly predicted the average indoor PM_2.5_ concentration on one or more days, and do not adequately account for the fluctuation of indoor PM_2.5_ concentration during the day (or longer) and the variability of individual behaviors over time (19–21). Obviously, the establishment of indoor PM_2.5_ concentration prediction models with higher temporal resolution is of more practical significance to improve individual PM_2.5_ exposure assessment. The existing models were constructed using indoor PM_2.5_ concentration monitoring data from a single type of place, which is not universal enough and inevitably limits the practical application to different types of places. No study has yet established prediction models for hourly average indoor PM_2.5_ concentration based on data from multiple types of places.

In this study, monitored data on indoor PM_2.5_ concentrations from five types of typical sites (offices, primary and secondary schools, kindergartens, shopping malls, and restaurants) in Shanghai were collected during different seasons. The data were used to develop and evaluate predictive MLR and RFR models for indoor PM_2.5_ temporal average concentrations based on multiple types of places. The aim of the study was to provide a feasible way to improve individual PM_2.5_ exposure assessment.

## 2. Materials and methods

### 2.1. Data collection

Five types of typical locations – offices, middle and primary schools, kindergartens, shopping malls, and restaurants – were selected for indoor PM_2.5_ concentration field monitoring in 16 districts of Shanghai. A TSI DustTrak 8,530 benchtop aerosol monitor (TSI Incorporated, Shoreview, MN, United States) was used for the monitoring. One floor was selected as the monitoring site for the high, middle, and low areas of office buildings, shopping malls, and restaurants. Two, four, and six monitoring points were set for indoor areas of 200–1,000 m^2^, 1,001–5,000 m^2^, and over 5,000 m^2^, respectively. Two classrooms from each floor were used as monitoring sites in high, middle, and low areas of kindergartens, middle, and primary schools. One, three, and five monitoring points were set for indoor areas of less than 50 m^2^, 50–100 m^2^, and more than 100 m^2^, respectively. All of the above points were distributed evenly on the diagonal of the room or in a plum style, and the height of each point was set at the level of a human respiratory belt (0.8–1.2 m). The actual measurement time was in January, April, July, and October of 2018 (the 4 months represented the four seasons of the year: January for winter, April for spring, July for summer, and October for autumn). Indoor PM_2.5_ concentrations in each location were monitored for 1 week during these 4 months, with each instrument monitoring the concentrations every 15 min, which covered all times of the day (00,00–23,00 h) to ensure full coverage of people’s activities in various places as much as possible.

For the construction of prediction models, we used the findings of relevant publications (17, 21–24) to identify 11 easily accessible indicators that may have significant effects on indoor PM_2.5_ concentrations. The relevant information of the indicators could be found in Supplementary Table S1. The outdoor PM_2.5_ and PM_10_ concentration data were obtained from the monitoring stations of 16 municipal control points in Shanghai. By calculating the distance between all government-controlled monitoring stations and the indoor places we monitored, the data from the closest station was selected as outdoor PM_2.5_ and PM_10_ concentration data for indoor places. Meteorological data for the same period were obtained from the European Center for Medium and Long-Range Weather Forecasts, which included outdoor temperature, relative humidity, air pressure, precipitation, surface wind speed, and wind direction.

### 2.2. Data analysis

The data analysis in this study was based on the arithmetic mean of time, that is, the indoor and outdoor PM_2.5_ concentrations, outdoor PM_10_ concentration, as well as related meteorological parameters were processed as hourly mean values for use. For example, the indoor PM_2.5_ concentration at 09:00 h was actually the mean value of 08:00 h to 09:00 h. Following a series of data washing, the final database consisted of 11,712 records, 11 potential predictor variables, and natural log-transformed indoor PM_2.5_ concentrations (approximately normally distributed) as response variables for MLR and RFR model construction. Data analysis and model construction in this study were performed with R software (version 4.1.0), and statistical significance levels were set at *p* values of <0.01 and < 0.05 (both sides).

### 2.3. MLR model construction steps

A sensitivity analysis was conducted for the effects of different variable screening methods on the predictive efficacy of MLR models. The three adopted types of variable screening were as follows: 1) manually supervised forward linear regression commonly used in reference to classical land-use regression modeling (25, 26), 2) stepwise regression (backward, variables with regression coefficient *p* < 0.05 were retained), and 3) least absolute shrinkage and selection operator (Lasso). The manually supervised forward linear regression method was used to build a basal multiple regression model in three steps: 1) After testing the premise assumptions of the regression model, all potential predictor variables expected to be included in the model were first univariately regressed against the response variable (natural log-transformed hourly average PM_2.5_ concentration), and predictor variables with significant (*p* < 0.05) regression coefficients were retained for the next step, 2) Correlations between prediction variables were tested. Among the prediction variables that were highly correlated with other prediction variables (Spearman r > 0.50, *p* < 0.05), only the prediction variable with the highest coefficient of determination (R^2^) was retained for further analysis, 3) The predictor variables that remained after the previous two steps were sorted according to R^2^ (from highest to lowest), and then each predictor was entered into the regression model in order. Finally, only those predictor variables with significant partial regression coefficients (*p* < 0.05), which boosted the R^2^ of the model by more than 1% and whose coefficients were consistent with the priori hypothesis (such as a positive coefficient of outdoor PM_2.5_), were retained.

In the process of MLR model diagnosis, variance inflation factors of the predictive variables were tested to evaluate multicollinearity. Additionally, considering that season may modify the effects of other potential predictor variables on indoor PM_2.5_ concentration, we stratified the data by winter–spring (January, April) and summer-autumn (July, October) seasons and developed season-specific prediction models.

### 2.4. RFR model construction steps

Random forest model is a machine learning model that realizes the classification and/or prediction for unknown samples through the integrated learning with a large number of decision trees, which is now widely used in the processing of big data due to its fast computing speed, high prediction accuracy, and strong anti-interference (27–29). This model possesses two significant characteristics, namely sample randomization and variable randomization. Bagging algorithm is the basis of the random forest model, which is also known as bootstrap sampling algorithm, in short, there is put back to the random collection of samples to form a different set of data to train the base learner, so as to realize the mutual independence of individual learners. The Random Forest algorithm extends and expands the Bagging algorithm. In addition to random sampling of samples, the Random Forest algorithm also incorporates random selection of variables at each attribute node of the classification tree, which further enhances the diversity of each decision tree, reduces the risk of model overfitting, and can effectively improve the generalization performance of the final ensemble model (27, 29). The prediction accuracy and generalization of a Random Forest model are closely related to two important hyperparameters, which are ntree (the number of trees used) and mtry (the number of variables used for binary trees in the specified nodes). The randomForest package of R software (version 4.1.0) was used to construct the RFR model. In our analysis, different values were set for these two parameters as sensitivity analysis in order to obtain maximum model prediction effectiveness. The increase in mean squared error (%IncMSE) of the predicted value was taken as an indicator to measure the importance of a variable, in other words, a random value was assigned to each prediction variable. If the prediction variable is important, the prediction error of the model will increase after its value is randomly replaced, so the larger the value, the more important the variable is.

In order to evaluate and compare the prediction efficiency of the MLR model and the RFR model for indoor hourly average PM_2.5_ concentration in various types of places, we developed two RFR models. The first RFR model was called the Full variables-RFR model (Full-RFR). Since the RFR model does not need to consider preconditions such as the independence of predictive variables that are faced by general MLR models, all 11 potential predictive variables were included in the model. The second RFR model was called the Conjoint-RFR model (Conjoint-RFR). In order to compare the MLR and RFR models, this Conjoint-RFR model was established using the same predictor variables as the MLR model with the best prediction performance identified in the previous steps.

### 2.5. Evaluation of models

The R^2^ and root mean squared error (RMSE) calculated based on the predicted and measured values of the model were used as the model performance evaluation indexes. In addition, the generalization performance of the model was evaluated by a ten-fold cross-validation (CV) method. In short, the entire dataset was randomly and equally divided into ten subsets, nine of which were selected as the training set and the remaining one was used as the test set to test the prediction performance of the model. This process was repeated 10 times until each subset was used for one verification (30).

## 3. Results

### 3.1. Indoor PM_2.5_ pollution in various places

**Figure 1:**
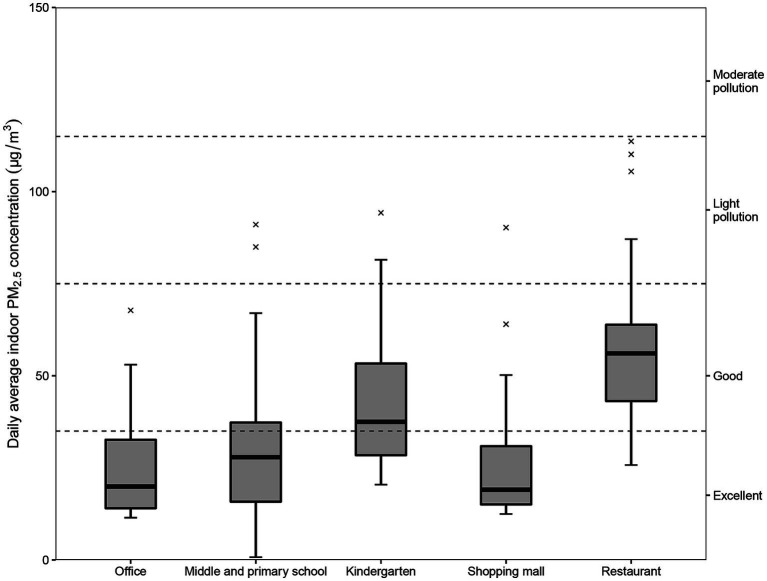
Daily average indoor PM_2.5_ concentrations (μg/m^3^) in each place. In reference to the classification method of the China Environmental Monitoring Station: 0–35 μg/m^3^ is excellent; 35–75 μg/m^3^ is good; 75–115 μg/m^3^ is light pollution; and 115–150 μg/m^3^ is moderate pollution. PM_2.5_ refers to particulate matter with an aerodynamic diameter of 2.5 μm or less.

The summary of hourly average indoor PM_2.5_ concentration statistics for each site was shown in Table 1. In general, the median hourly average indoor PM_2.5_ concentration was 34.9 μg/m^3^ and the interquartile range was 24.5 μg/m^3^, with a few readings on the high side and a maximum value of 288 μg/m^3^. The result of Welch analysis of variance (Welch ANOVA) (31) showed significant differences (*p* < 0.01) in the hourly average indoor PM_2.5_ concentrations in different types of places. The highest hourly average indoor PM_2.5_ concentrations were found in restaurants (44.4 μg/m^3^), probably because of frequent cooking in restaurants that produces a large amount of grease smoke and causes indoor PM_2.5_ concentrations to increase (32). The Ambient Air Quality Standards (GB 3095–2012) of China and the Environmental Protection Agency of the United States have set the daily average ambient PM_2.5_ concentration limit at 35 μg/m^3^. No clearly established indoor PM_2.5_ concentration standard exists in China; therefore, the daily average ambient PM_2.5_ concentration standard and the classification method of the China Environmental Monitoring Station were used here to characterize the indoor PM_2.5_ pollution in each location (Figure 1). In terms of 35 μg/m^3^ as the standard, indoor PM_2.5_ exceeded the standard in different degrees in all places and restaurants were the worst offender, followed by kindergartens. The monitoring results suggest that the indoor environmental quality of these two types of places needs to be improved.

**Table 1:** Hourly average indoor PM_2.5_ concentrations in each place (μg/m^3^).

Type of place	*n*	Arithmetic mean	SD	Percentiles				
				Min	P25	P50	P75	Max
Office	3,438	23.8	15.6	0.667	12.0	18.8	32.5	128
Middle and primary school	1,812	33.5	20.2	0.20	20.2	30.9	43.1	127
Kindergarten	1,441	37.6	19.8	5.19	24.2	34.3	45.9	154
Shopping mall	2,443	31.2	15.8	4.0	20.0	28.8	39.5	133
Restaurant	2,578	52.6	34.2	4.86	28.3	44.4	67.2	288
Overall	11,712	34.9	24.5	0.2	17.7	30.1	44.1	288

*n*, the number of samples; SD, standard deviation; Min, the minimum value; P25, P50, and P75: the 25th, 50th, and 75th percentiles, respectively; Max, the maximum value; PM_2.5_ refers to particulate matter with an aerodynamic diameter of 2.5 μm or less.

**Figure 2:**
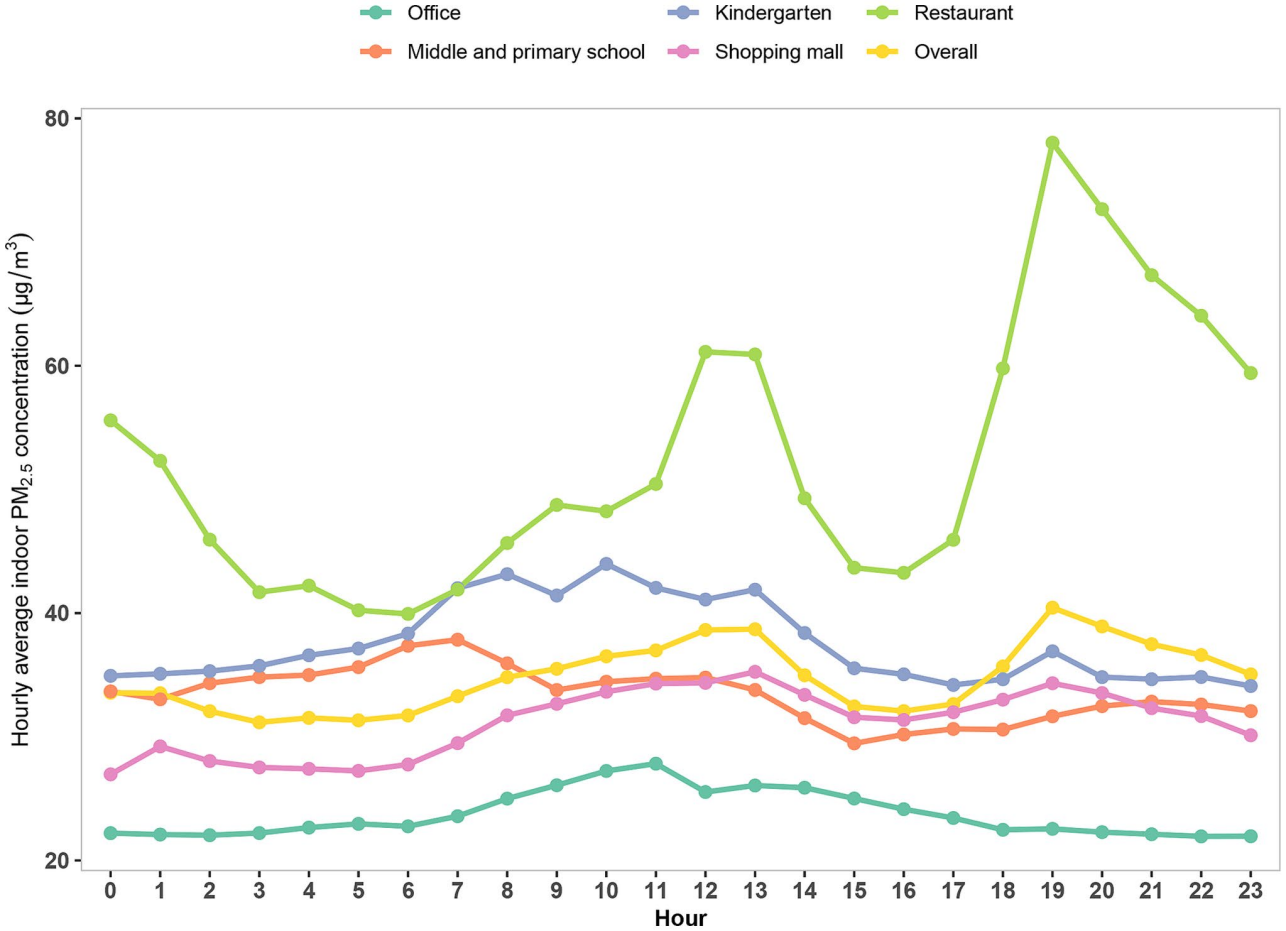
Variation of intraday hourly average indoor PM_2.5_ concentration in each place (μg/m^3^). PM_2.5_ refers to particulate matter with an aerodynamic diameter of 2.5 μm or less.

The changes of hourly average indoor PM_2.5_ concentration at different times are shown in Figure 2. Overall, there were significant differences (*p* < 0.01) in indoor PM_2.5_ at different times of the day, and we also observed significant intraday fluctuations in the monitoring data for each type of place (*p* < 0.05). The variability of PM_2.5_ concentration at different times of the day in multiple types of places is closely related to the nature of the place. For example, the fluctuation of PM_2.5_ concentration in the restaurant was as expected (*p* < 0.01), with two peaks occurring after 11:00 and after 17:00, which are roughly the beginning of lunch and dinner. At these times, intensive cooking leads to higher indoor PM_2.5_ concentrations, and similar patterns were observed in other places (Figure 2). These results demonstrate the intraday variability of indoor PM_2.5_ concentration as well as the spatial variability across places.

### 3.2. MLR model results

**Figure 3:**
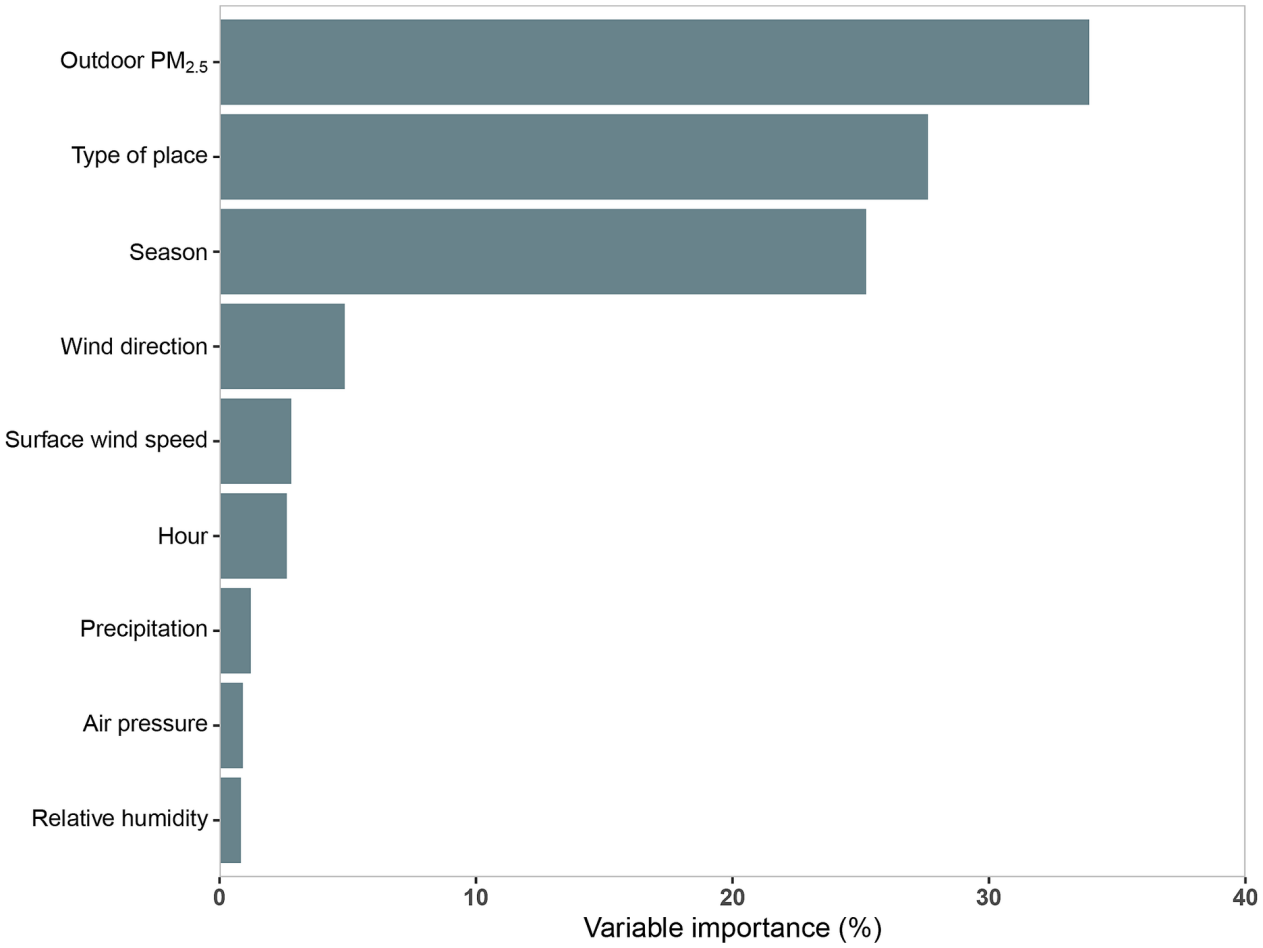
Relative importance of the multiple linear regression (MLR) model predictor variables. R^2^, coefficient of determination; PM_2.5_ refers to particulate matter with an aerodynamic diameter of 2.5 μm or less.

Univariate regression model results for hourly average indoor PM_2.5_ concentration were summarized in Supplementary Table S2. All 11 prediction variables were significantly associated with hourly average indoor PM_2.5_ (*p* < 0.05). The R^2^ of the 11 prediction variables ranged from 0.1 to 30.54%, among which nine variables exceeded 2%, with the largest R^2^ for outdoor PM_2.5_ concentration (30.54%), followed by outdoor PM_10_ concentration (R^2^ = 28.76%), season (R^2^ = 24.05%), type of place (R^2^ = 17.11%), and wind direction (R^2^ = 8.64%). The final MLR model for log-transformed hourly average indoor PM_2.5_ concentrations were shown in Table 2. The model which was developed based on the stepwise regression method had the best prediction performance (CV R^2^ = 60.48%) and the lowest prediction error (CV RMSE = 0.44) among the three MLR models (Table 3). In this paper, the relative importance of the predictor variables within MLR model was determined using the “Lindeman, Merenda and Gold (LMG).” LMG was evaluated as the most successful indicator of the relative importance of independent variables, which was implemented by using the “relaimpo” package of R software (33, 34) (Figure 3). Outdoor PM_2.5_ concentration was the most important predictor variable, with an R^2^ share of 33.91%, followed by type of place (27.62%), season (26.22%), wind direction (4.88%), and surface wind speed (2.80%). The two models developed after stratification by winter–spring and summer-autumn incorporated similar predictor variables, of which the R^2^ and RMSE after cross-validation were also remarkably close (winter–spring model: R^2^ = 58.23%, RMSE = 0.38; summer-autumn model: R^2^ = 58.79%, RMSE = 0.49; Supplementary Tables S3–S5).

**Table 2:** Multiple linear regression (MLR) model for log-transformed hourly average indoor PM_2.5_.

Predictive variables	Coefficients	Standard error	*p*-value	Partial R^2^ (%)
*Intercept*	17.60	1.73	**<0.01**	
*Outdoor PM_2.5_*	0.014	0.013	**<0.01**	33.91
*Type of place*	—	—	—	27.62
Office (reference)	—	—	—	
Middle and primary school	0.035	0.014	**<0.01**	
Kindergarten	0.23	0.015	**<0.01**	
Shopping mall	0.16	0.012	**<0.01**	
Restaurant	0.76	0.011	**<0.01**	
Season	—	—	—	26.22
Winter (reference)	—	—	—	
Spring	0.015	0.027	0.58	
Summer	−0.73	0.037	**<0.01**	
Autumn	−0.03	0.022	0.14	
*Wind direction*	0.00024	0.000062	**<0.01**	4.88
*Surface wind speed*	−0.008	0.0024	**<0.01**	2.80
*Hour*	—	—	—	2.62
0 (reference)	—	—	—	
1	−0.024	0.028	0.39	
2	−0.06	0.028	**<0.05**	
3	−0.10	0.028	**<0.01**	
4	−0.12	0.029	**<0.01**	
5	−0.13	0.029	**<0.01**	
6	−0.12	0.029	**<0.01**	
7	−0.06	0.029	**<0.05**	
8	−0.018	0.029	0.51	
9	0.043	0.028	0.13	
10	0.069	0.028	**<0.05**	
11	0.10	0.028	**<0.01**	
12	0.16	0.028	**<0.01**	
13	0.17	0.028	**<0.01**	
14	0.16	0.028	**<0.01**	
15	0.095	0.028	**<0.01**	
16	0.092	0.028	**<0.01**	
17	0.10	0.028	**<0.01**	
18	0.16	0.028	**<0.01**	
19	0.21	0.028	**<0.01**	
20	0.16	0.028	**<0.01**	
21	0.10	0.028	**<0.01**	
22	0.076	0.028	**<0.01**	
23	0.016	0.028	0.09	
*Precipitation*	−0.19	0.012	**<0.01**	1.22
*Air pressure*	−0.015	0.0012	**<0.01**	0.91
*Relative humidity*	0.26	0.042	**<0.01**	0.83

Significant *p*-values are in bold. PM_2.5_ refers to particulate matter with an aerodynamic diameter of 2.5 μm or less; R^2^, coefficient of determination.

**Table 3:** Summary of model performance evaluation results.

Models	Model-based indicators		Ten-fold cross-validation indicators	
	Coefficient of determination (R^2^, %)	Root mean square error (RMSE)	Coefficient of determination (R^2^, %)	Root mean square error (RMSE)
Basal MLR model	59.51	0.44	59.38	0.45
MLR with lasso selection	60.54	0.43	60.35	0.45
MLR with stepwise selection	60.67	0.43	60.48	0.44
Conjoint-RFR model	89.65	0.23	71.86	0.37
Full-RFR model	91.20	0.21	72.20	0.36

The multiple linear regression (MLR) models were developed by three different variable selection methods. Two random forest regression (RFR) models were developed using 200 trees.

### 3.3. RFR model results

**Figure 4:**
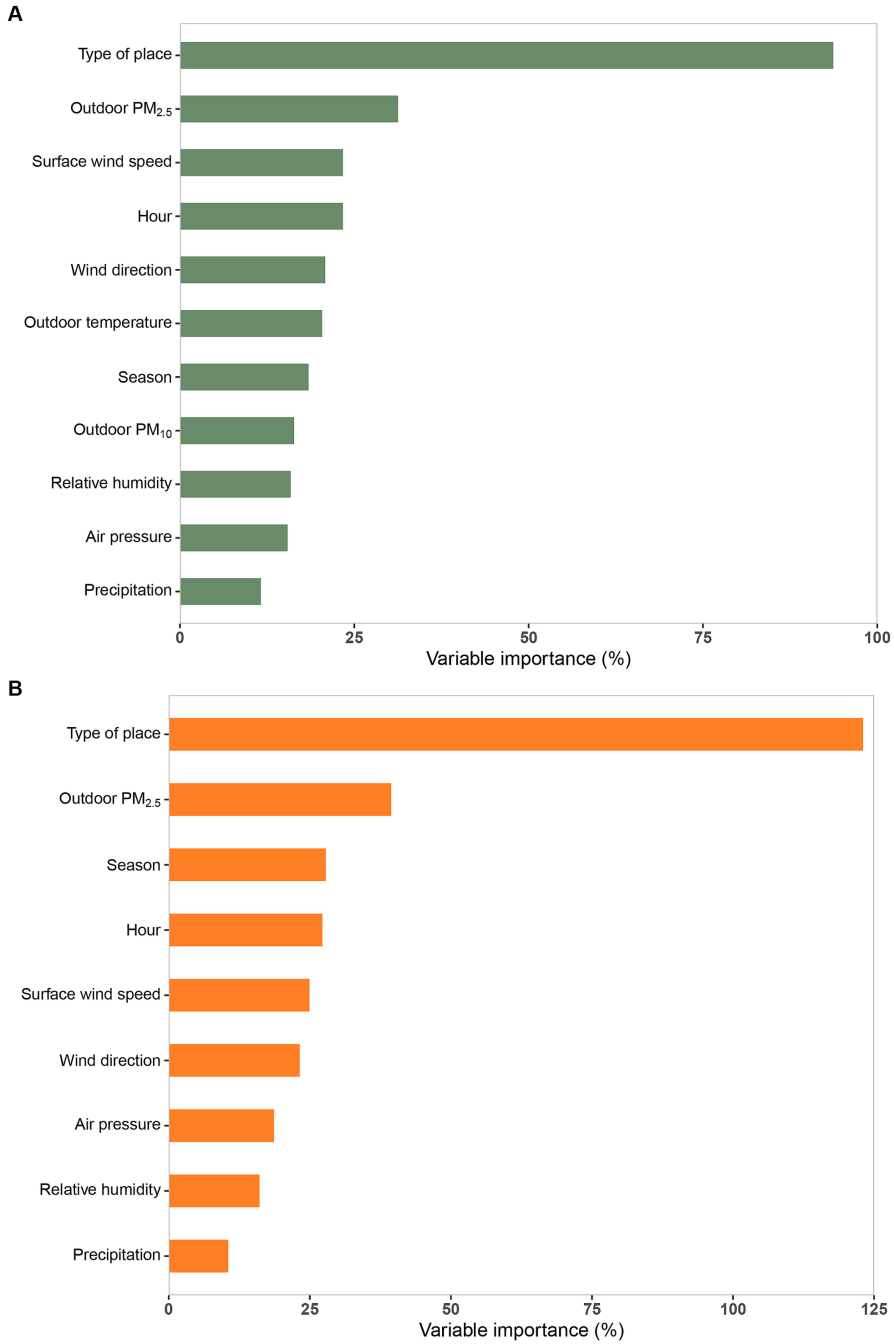
The importance of the predictor variables in random forest regression (RFR) models based on “%IncMSE.” Full-RFR model **(A)**, Conjoint-RFR model **(B)**. PM_2.5_ and PM_10_ refer to particulate matter with an aerodynamic diameter of 2.5 μm or less and of 10 μm.

We compared and analyzed all RFR models with ntree of 200, 500, 1,000 and mtry of 1 ~ 11 (Supplementary Figure S1), and finally determined that ntree = 200 and mtry = 2 were the most suitable RFR parameters for this study after fully considering the model’s prediction effectiveness, prediction error, and model efficiency. Results from the Conjoint-RFR model, which used the same predictor variables as the MLR model, showed that the RFR model explained a greater proportion of the variance of indoor PM_2.5_ time-averaged concentrations with an R^2^ (RMSE) of 89.65% (0.23), which decreased in predictive efficacy (CV R^2^ = 71.86%) and increased in prediction error (CV RMSE = 0.37) after ten-fold cross-validation. Nevertheless, the overall performance of the model was still better than that of the corresponding MLR model (CV R^2^ = 60.48; CV RMSE = 0.44). The performance of the Full-RFR model incorporating all predictor variables was better than that of the Conjoint-RFR model, with a CV R^2^ (RMSE) of 72.20% (0.36). The importance results of the predictor variables from the random forest algorithm (Figures 4A,B) indicated that the top five variables in the Conjoint-RFR model (Figure 4B) in order of importance were type of place, outdoor PM_2.5_ concentration, season, hour, and surface wind speed. Comparison of the importance ranking results of the variables in the Conjoint-RFR model and the corresponding MLR model shows that the top three variables in both models are the same, namely, outdoor PM_2.5_ concentration, type of place, and season, but with a different order. By contrast, the variable “hour” appears in the top five variables in the Conjoint-RFR model but wind direction is in the top five in the MLR model.

## 4. Discussion

Significant differences in indoor PM_2.5_ concentrations between various types of places and at different times of day were found in our study. The variable of “type of place” ranked first and second in the importance assessment of the predictor variables of the RFR model and the MLR model in this study, respectively. This result emphasized the importance of place type in predicting indoor PM_2.5_ concentration and suggested that it might be difficult to extrapolate the prediction model based on a single type of place for use in other types of places. In fact, it is not difficult to understand the conclusion that the different functional attributes of each place naturally create a unique indoor microenvironment, which consequently affects the occurrence, diffusion, deposition and other behaviors of PM_2.5_ (35–38). For example, in an office, there is a high concentration of people, frequent use of office equipment (e.g., printers, photocopiers and computers), and air-conditioning equipment (air-conditioners, humidifiers, air filters), with low ventilation and a single source of indoor pollution, whereas in a shopping mall there is a higher flow of people, more frequent ventilation, and a more complex internal environment. In contrast, the frequent cooking activities in restaurants generate smoke and high temperatures, creating a different microenvironment than the places mentioned above (35, 39). However, the currently available prediction models for indoor PM_2.5_ concentrations are all constructed based on monitoring data from a single type of place, such as residential buildings (16, 18, 40, 41), schools (19, 20), and offices (42), without considering the differences between various types of sites. This situation inevitably leads to limitations in the actual application for the assessment of indoor PM_2.5_ exposure. At present, no study has attempted to construct an indoor PM_2.5_ concentration prediction model based on monitoring data from multiple types of places, and our study has attempted to fill this gap. In addition, most existing studies have predicted indoor PM_2.5_ concentration over a day or longer period (such as a week); however, many published studies have shown that indoor PM_2.5_ concentrations have a large daily variability (19–21). According to a report by Che et al. (43), after conducting continuous monitoring of indoor air quality in 32 primary and secondary schools across Hong Kong, it was found that there were significant variations in PM_2.5_ concentrations in classrooms at different times of the day. The PM_2.5_ concentrations in classrooms during school hours were approximately 40% higher than non-school hours. Zhao et al. (44) reported that indoor PM_2.5_ concentrations were 1.5 times higher at night than during the daytime in Beijing during winter. According to Xu et al. (13), indoor PM_2.5_ concentrations at different moments of the day varied significantly, with the ratio of the highest to the lowest values even exceeding 15-fold. This temporal variability of indoor PM_2.5_ may originate from outdoor sources, for example, factors such as changes in outdoor PM_2.5_ concentrations, variations in wind direction, temperature, and atmospheric pressure throughout the day and night may contribute to the differences in indoor PM_2.5_ concentrations (17, 44), or from indoor human activities, such as cooking, smoking, use of air purifiers, etc. (45, 46). No matter what causes this variability, establishing a higher temporal resolution in an indoor PM_2.5_ concentration prediction model is more practical for refining individual PM_2.5_ exposure assessment and health risk evaluation.

MLR models are widely used for indoor air quality prediction because of the advantages of simple methodology, easy application, and strong interpretation of results (13, 17, 47). However, prerequisites exist for MLR application. First, a linear relationship must exist between the prediction variable and the response variable. Second, the response variable must obey a normal distribution when each predictor variable takes a certain definite value. Third, the response variable must satisfy the homogeneity of variance when each predictor variable takes different values. Fourth, the predictor variables are independent of each other and do not have a very close statistical correlation. These prerequisites for MLR in practical applications are sometimes not easily satisfied.

With improvements in computing power and the advent of the era of big data, machine learning algorithms have been constantly enhanced and widely focused. The random forest algorithm is an integrated decision tree-based algorithm proposed by Breiman and Cutler in 2001, which can simultaneously construct a large number of decision trees in parallel and achieve significantly higher computational efficiency than other machine learning methods by integrating the learning of multiple decision trees (27, 29). Due to the inherent inclusion of interactions between variables in the random forest algorithm, there is no need to consider the issue of multicollinearity among variables in general models, and the algorithm performs robustly with mixed data types, missing data, non-equilibrium data, and extreme data, leading to a high prediction accuracy of the model (28). In addition, owing to the inclusion of sample perturbation and attribute perturbation in the algorithm, the random forest model can effectively limit overfitting and is regarded as one of the best algorithms today (48–50). Of course, random forest models also have certain drawbacks, such as poor interpretability of the model, which is usually considered as a black box model. Furthermore, categorical variables with more levels will have a greater impact on the model results than those with fewer levels, which may lead to a deviation in the prediction results (48, 51).

In our study, MLR and RFR prediction models were developed for hourly average indoor PM_2.5_ concentrations based on monitoring data from multiple types of places. As a conventional and classical prediction model, the MLR model is widely used to predict indoor PM_2.5_ concentration. Our MLR model (CV R^2^ = 60.48%) had a relatively high predictive performance compared with published MLR prediction models of indoor PM_2.5_ concentration based on 1 day or longer (such as 1 week) whose R^2^ values ranged from 33 to 87% (13, 16, 18, 19, 52–54). To the best of our knowledge, only one study by Xu et al. (13) has developed an MLR prediction model for hourly average indoor PM_2.5_ concentration. In this study, two MLR models were developed for two regions with CV R^2^ values of 71 and 75%.

The two CV R^2^ values in the study by Xu et al. (13) indicated better model predictive performance than for our MLR model. This difference might be because the model development in our paper was based entirely on easily accessible temporal indicators and outdoor indicators. By contrast, the model construction in the study by Xu et al. (13) incorporated not only outdoor indicators (such as outdoor PM_2.5_ concentration and outdoor relative humidity) but also indoor indicators (such as indoor smoking and cooking), with a wide range of indicator coverage. However, the model in that study also suffered from difficulties in the definition of relevant indicators, such as “whether or not to cook.” In fact, cooking ingredients, cooking methods, cooking time, and the type of oil used have significant effects on indoor PM_2.5_ concentration (55, 56). Moreover, these types of prediction indicators were not easy to obtain and the process was costly. Only several studies have developed RFR prediction models for indoor PM_2.5_ concentration, and the CV R^2^ values have ranged from 48.9 to 82% in these studies (13, 16, 18). The predictive efficacy of the Full-RFR model in this study (CV R^2^ = 72.20%) was also at a high level.

MLR and RFR models, as common indoor PM_2.5_ concentration prediction models, are still controversial in terms of which approach can better predict indoor PM_2.5_ concentrations. Previous studies have shown (16, 44) that using the same dataset, an RFR model usually outperforms an MLR model in terms of predictive efficacy owing to the strength of the algorithm itself, such as robustness to missing data and good characterization of interactions between different predictor variables. However, some studies have reached the opposite conclusion, as in the study by Yuchi et al. (18). In their study, two models had the same variables for the same dataset, and the MLR model (CV R^2^ = 50.2%) outperformed the RFR model (CV R^2^ = 48.9%) in terms of generalization performance. This issue was also explored in the current study, as the results of our sensitivity analysis for the modeling algorithm showed that the Full-RFR model, which used all predictor variables, and the Conjoint-RFR model, which used the same predictor variables as MLR, both performed better than the MLR model.

Compared with other studies, the current study had several strengths. First, the indoor PM_2.5_ concentration monitoring data based on multiple types of places were used for modeling, which was more generalizable for predicting indoor PM_2.5_ concentration than the models developed using data from a single type of place. Second, we developed modeling with high temporal resolution indoor PM_2.5_ concentration data (hourly average data), which fully took into account the temporal variability of indoor PM_2.5_. Third, the sample size used for modeling was sufficiently large (*n* = 11,712) to greatly exceed the number of predictor variables (11), so that the model was less prone to overfitting. Fourth, the model prediction cost was low, and the predictor variables in the model were all easy to obtain. For example, outdoor PM_2.5_ concentration, wind direction, and surface wind can be found through the websites of relevant government departments. The model is suitable for epidemiological studies with large populations and/or long time periods.

Of course, there were some limitations in the study. First, the outdoor PM_2.5_ concentration data of indoor places in the study were obtained from the nearest government-controlled monitoring sites. Although this approach has been used in many previous studies, it could introduce some errors in the model due to the spatial variability of outdoor PM_2.5_ concentrations. Second, the absence of human indoor activity variables, such as smoking and cooking, might cause an increase in the prediction error of the model at certain time periods and contexts, for instance, during cooking and when air purifiers were used. Third, the model was developed and evaluated based on data from Shanghai, and there was a lack of equivalent data from other regions for further validation of model performance.

## 5. Conclusion

We found significant differences in indoor PM_2.5_ concentration between types of places and time periods. This finding reflects the possible limitations of models based on indoor PM_2.5_ concentration data from a single type of place as well as the necessity for a prediction model with a high temporal resolution in order to perfect individual PM_2.5_ exposure assessment. Here, we aimed to develop MLR and RFR models for hourly average indoor PM_2.5_ concentration over multiple types of places. Both statistical models were based on easy-to-access indicators and showed good predictive efficacy. They could, therefore, be used for quantitative estimation of indoor PM_2.5_ exposure in large-scale population studies. In addition, the performance of the classical MLR model and machine learning RFR model were evaluated comparatively in predicting indoor PM_2.5_ concentration, and the model performance metrics showed that the RFR model using the same dataset outperformed the MLR model. This finding suggests the potential of RFR models in predicting indoor air pollutant levels, and other machine learning algorithms may also be worthy of exploration.

## Data availability statement

The raw data supporting the conclusions of this article will be made available by the authors, without undue reservation.

## Author contributions

YS, ZD, CD, and SS designed the research. YS, FH, FC, DW, ML, and HZ performed data acquisition, organization and a part of analysis. ZD and YS analyzed the data and wrote the primary manuscript. YS, ZD, CD, SS, FH, and FC provided a contribution to the explanation of the findings and critically reviewed and edited the manuscript. All authors contributed to the article and approved the submitted version.

## Funding

This work was supported by Shanghai Municipal Health Commission Science and Research Fund (No. 202040185).

## Conflict of interest

The authors declare that the research was conducted in the absence of any commercial or financial relationships that could be construed as a potential conflict of interest.

## Publisher’s note

All claims expressed in this article are solely those of the authors and do not necessarily represent those of their affiliated organizations, or those of the publisher, the editors and the reviewers. Any product that may be evaluated in this article, or claim that may be made by its manufacturer, is not guaranteed or endorsed by the publisher.
