# Supplementary material for: Construction and evaluation of hourly average indoor PM2.5 concentration prediction models based on multiple types of places
Source: Front Public Health. 2023 Aug 10;11:1213453. doi: 10.3389/fpubh.2023.1213453 (PMC10447970; doi:10.3389/fpubh.2023.1213453)
Supplement: Supplementary file 1 [file Data_Sheet_1.pdf]

Supplementary Material

Construction and evaluation of hourly average indoor PM2.5 concentration prediction models based on multiple types of places

Table S1

The list of candidate predictors for hourly average indoor PM2.5

Variable	Description	Type	Value/Unit
**Type of place**	Office; middle and primary school; kindergarten; shopping mall; restaurant	Categorical variable	Office =1；Middle and primary school =2；Kindergarten =3；Shopping mall =4；Restaurant =5
**Season**	Spring (January); summer (April); autumn (July); winter (October)	Categorical variable	Spring=1; summer=2; autumn=3; winter =4
**Hour**	00:00–23:00	Categorical variable	0，1，2，3……23
**Outdoor PM2.5 concentration**	PM2.5 concentration data from the nearest municipal control monitoring station to the indoor place	Continuous variable	μg/m^3
**Outdoor PM10 concentration**	PM10 concentration data from the nearest municipal control monitoring station to the indoor place	Continuous variable	μg/m^3
**Outdoor temperature**	The temperature in the louvered box at a height of about 1.5–2 m above the ground	Continuous variable	℃
**Relative humidity**	Relative humidity of air about 1.25 to 2 m above ground	Continuous variable	%
**Surface wind speed**	Wind speed of about 10 m above the ground	Continuous variable	m/s
**Air pressure**	Air pressure values in the region	Continuous variable	hPa
**Precipitation**	It is the depth of liquid or solid water (after melting) that falls from the sky to the ground and accumulates on the horizontal surface without evaporation, infiltration, or loss	Continuous variable	mm/h
**Wind direction**	The direction of the incoming wind, due north is 0°, clockwise is positive	Continuous variable	°

Table S2

One-independent variable regression models for predictors of log-transformed hourly indoor PM2.5. Significant p-values are in bold.

Predictive variables	Coefficients	Standard error	R^2 (%)
**Outdoor PM2.5**	0.018	**<0.01**	30.54
**Outdoor PM10**	0.012	**<0.01**	28.76
**Season**	—	—	24.05
Winter (reference)	—	—	
Spring	0.36	**<0.01**	
Summer	-0.49	**<0.01**	
Autumn	0.31	**<0.01**	
**Type of place**	—	—	17.11
Office (reference)	—	—	
Middle and primary school	0.29	**<0.01**	
Kindergarten	0.53	**<0.01**	
Shopping mall	0.35	**<0.01**	
Restaurant	0.78	**<0.01**	
**Wind direction**	0.0023	**<0.01**	8.64
**Surface wind speed**	-0.083	**<0.01**	5.15
**Outdoor temperature**	-0.012	**<0.01**	2.65
**Relative humidity**	-0.64	**<0.01**	2.07
**Hour**	—	—	1.28
0 (reference)	—	—	
1	-0.0059	0.89	
2	-0.034	0.44	
3	-0.057	0.19	
4	-0.064	0.15	
5	-0.082	0.06	
6	-0.070	0.11	
7	-0.018	0.69	
8	0.036	0.42	
9	0.072	0.10	
10	0.10	**<0.05**	
11	0.13	**<0.01**	
12	0.17	**<0.01**	
13	0.19	**<0.01**	
14	0.14	**<0.01**	
15	0.059	0.18	
16	0.035	0.44	
17	0.053	0.23	
18	0.091	**<0.01**	
19	0.16	**<0.01**	
20	0.13	**<0.01**	
21	0.10	**<0.05**	
22	0.079	0.07	
23	0.044	0.32	
**Precipitation**	-0.13	**<0.01**	0.53
**Air pressure**	0.0097	**<0.01**	0.10

Table S3

Final winter-spring multiple linear regression (MLR) model for log-transformed hourly average indoor PM2.5. Significant p-values are in bold.

Predictive variables	Coefficients	Standard error	P-value
**Intercept**	16.583	0.668	**<0.01**
**Outdoor PM2.5**	0.0138	0.000224	**<0.01**
**Type of place**	—	—	—
Office (reference)	—	—	—
Middle and primary school	0.193	0.0149	**<0.01**
Kindergarten	0.278	0.0137	**<0.01**
Shopping mall	0.215	0.0143	**<0.01**
Restaurant	0.788	0.0141	**<0.01**
**Precipitation**	-0.151	0.00981	**<0.01**
**Surface wind speed**	-0.0221	0.00252	**<0.01**
**Air pressure**	-0.0134	0.000656	**<0.01**
**Hour**	—	—	—
0 (reference)	—	—	—
1	-0.0513	0.0321	0.11
2	-0.0730	0.0321	**<0.05**
3	-0.100	0.0321	**<0.01**
4	-0.111	0.0322	**<0.01**
5	-0.121	0.0324	**<0.01**
6	-0.113	0.0322	**<0.01**
7	-0.0587	0.0322	0.068
8	-0.0410	0.0321	0.202
9	-0.00630	0.0319	0.844
10	-0.0076	0.0314	0.110
11	-0.00233	0.0313	0.941

Table S4

Final summer-autumn multiple linear regression (MLR) model for log-transformed hourly average indoor PM2.5. Significant p-values are in bold.

Predictive variables	Coefficients	Standard error	P-value
**Intercept**	2.697	0.0920	**<0.01**
**Outdoor PM2.5**	0.0122	0.000447	**<0.01**
**Type of place**	—	—	—
Office (reference)	—	—	—
Middle and primary school	-0.162	0.0230	**<0.01**
Kindergarten	0.0844	0.0486	0.0826
Shopping mall	0.0958	0.0194	**<0.01**
Restaurant	0.726	0.0180	**<0.01**
**Wind direction**	0.00167	0.000157	**<0.01**
**Precipitation**	0.945	0.182	**<0.01**
**Surface wind speed**	0.0309	0.00575	**<0.01**
**Relative humidity**	0.406	0.0686	**<0.01**
**Outdoor temperature**	-0.0401	0.00236	**<0.01**
**Hour**			
0 (reference)			
1	0.0205	0.0474	0.666
2	-0.0356	0.0475	0.454
3	-0.105	0.0476	**<0.05**
4	-0.142	0.0476	**<0.01**
5	-0.121	0.0477	**<0.05**
6	-0.0952	0.0477	**<0.05**
7	0.00832	0.0482	0.863
8	0.105	0.0484	**<0.05**

12	0.0524	0.0324	0.105
13	0.0831	0.0328	**<0.05**
14	0.0736	0.0324	**<0.05**
15	0.00182	0.0326	0.956
16	0.000125	0.0334	0.997
17	-0.00225	0.0332	0.946
18	0.0267	0.0326	0.412
19	0.0874	0.0326	**<0.01**
20	0.0496	0.0322	0.125
21	0.0239	0.0322	0.458
22	0.00741	0.0322	0.818
23	0.00509	0.0321	0.874

9	0.270	0.0506	**<0.01**
10	0.323	0.0480	**<0.01**
11	0.402	0.0489	**<0.01**
12	0.491	0.0504	**<0.01**
13	0.500	0.0507	**<0.01**
14	0.458	0.0505	**<0.01**
15	0.379	0.0494	**<0.01**
16	0.356	0.0488	**<0.01**
17	0.332	0.0473	**<0.01**
18	0.404	0.0471	**<0.01**
19	0.413	0.0469	**<0.01**
20	0.351	0.0471	**<0.01**
21	0.244	0.0469	**<0.01**
22	0.203	0.0467	**<0.01**
23	0.128	0.0467	**<0.01**

Table S5

Assessment results of model performance for the unstratified final multiple linear regression (MLR) model, winter-spring and summer-autumn MLR models.

Models	Model-based indicators		Ten-fold cross validation indicators	
	Coefficient of determination (R^2, %)	Root mean square error (RMSE)	Coefficient of determination (R^2, %)	Root mean square error (RMSE)
MLR with stepwise selection	60.67	0.43	60.48	0.44
Winter-spring MLR	59.34	0.38	58.23	0.38
Summer-autumn MLR	59.38	0.48	58.79	0.49

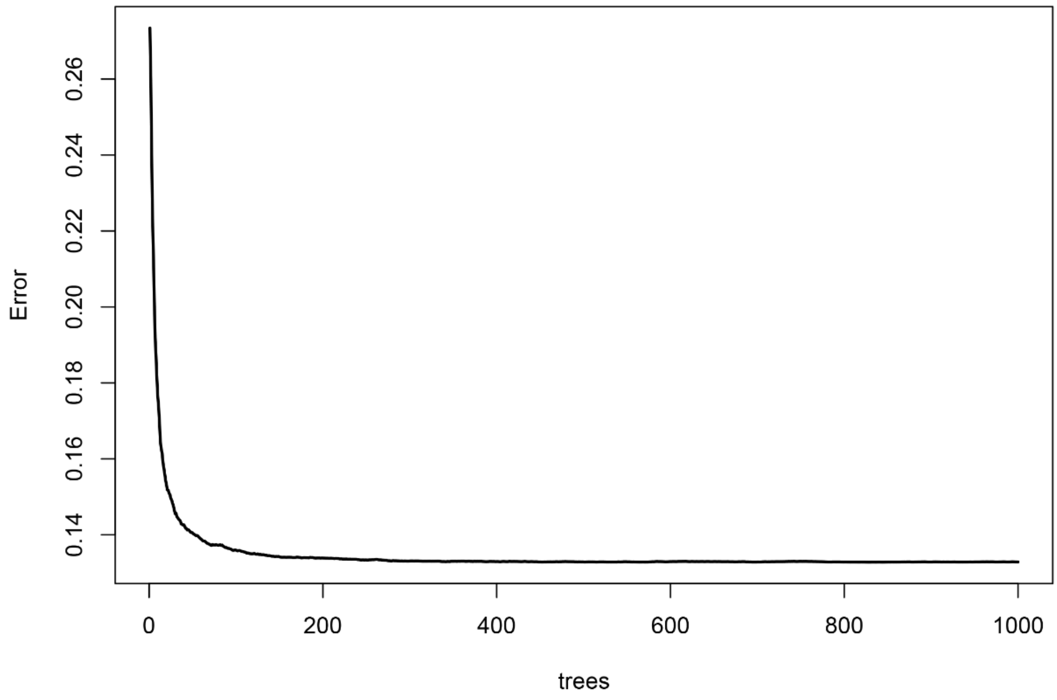

Figure. S1 Model error when setting different number of trees in RFR models
